# Combination of Three Functionalized Temperature-Sensitive Chromatographic Materials for Serum Protein Analysis

**DOI:** 10.3390/molecules24142626

**Published:** 2019-07-19

**Authors:** Weiwei Sun, Rongji Dai, Bo Li, Guoxin Dai, Di Wang, Dandan Yang, Pingping Chu, Yulin Deng, Aiqin Luo

**Affiliations:** 1 School of Life Science, Beijing Institute of Technology, Beijing 100081, China; 2 Advanced Research Institute of Multidisciplinary Science, Beijing Institute of Technology, Beijing 100081, China

**Keywords:** capture and release proteins, mouse serum, poly(*N*-isopropylacrylamide), functionalized temperature-sensitive chromatographic materials

## Abstract

We have developed a methodology to capture acidic proteins, alkaline proteins, and glycoproteins separately in mouse serum using a combination of three functionalized temperature-responsive chromatographic stationary phases. The temperature-responsive polymer poly(*N*-isopropylacrylamide) was attached to the stationary phase, silica. The three temperature-responsive chromatographic stationary phase materials were prepared by reversible addition–fragmentation chain transfer polymerization. Alkaline, acidic, and boric acid functional groups were introduced to capture acidic proteins, alkaline proteins, and glycoproteins, respectively. The protein enrichment and release properties of the materials were examined using the acidic protein, bovine serum albumin; the alkaline protein, protamine; and the glycoprotein, horseradish peroxidase. Finally, the three materials were used to analyze mouse serum. Without switching the mobile phase, the capture and separation of mouse serum was achieved by the combination of three temperature-responsive chromatographic stationary phase materials. On the whole, 313 proteins were identified successfully. The number of different proteins identified using the new method was 1.46 times greater than the number of proteins that has been identified without applying this method. To our knowledge, this method is the first combinatorial use of three functionalized temperature-responsive chromatographic stationary phase silica materials to separate proteins in mouse serum.

## 1. Introduction

Chromatographic techniques play an important role in the separation and purification of biological samples [1,2]. However, the separation mode of conventional high-performance liquid chromatography (HPLC) usually relies on changing the composition of the mobile phase to achieve good separation. This method takes a long time to balance the column. [3]. To address these aforementioned disadvantages, temperature-responsive chromatography has been developed. It is characterized by simple temperature control and separation conditions [4,5]. Separation of the analyte is achieved by controlling the temperature to change the surface properties of the stationary phase and increasing the selectivity of the column using the same mobile phase. Due to the reason that the temperature-responsive polymers exhibit temperature sensitivity in pure water or less-concentrated salt solutions, the mobile phase of temperature-responsive chromatography is typically pure aqueous phase solutions. The temperature of the stationary phase is generally controlled within the range of 10–50 °C, therefore the separation condition of temperature-responsive chromatography is very mild [6].

Poly(*N*-isopropylacrylamide) (PNIPAAm) is a typical temperature-responsive polymer with a low critical solution temperature (LCST) of 32 °C [7,8,9,10,11,12,13,14,15,16,17,18]. The molecular conformation and hydrophilic/hydrophobic properties of PNIPAAm exhibit sharp changes under thermal stimuli [19,20]. When the temperature is lower than the LCST, the polymer chain is highly stretched due to the hydrogen bonds formed between water molecules and the amide groups. However, when the temperature rises above the LCST, the polymer chain becomes crimped because hydrogen bonds cannot be formed between water molecules and isopropoxy groups. Moreover, copolymers can be formed by PNIPAAm and monomers of other ionizing groups. When PNIPAAm forms a copolymer with a hydrophobic monomer, the LCST value shifts to a lower temperature. On the other hand, the LCST value shifts to a higher temperature when PNIPAAm forms a copolymer with the ionic monomer. That is because the hydrogen bonding interaction can be enhanced between ionic monomers and water [21]. In 1992, PNIPAAm was grafted onto the surface of porous polymer beads and used as a gel permeation chromatography stationary phase to study the separation performance of dextran [22]. By controlling the temperature to change the pore size of the stationary phase, the retention time of dextran was significantly dependent on the temperature changes. In 1996, PNIPAAm was grafted onto aminopropyl silica by using an activated ester–amine coupling method [23]. The surface of these grafted material showed hydrophilic properties at lower temperatures, which will transform to hydrophobic surface properties as the temperature increases. Five mixed steroids can be separated by changing the temperature of the stationary phase on a HPLC column only. Since then, the types and applications of temperature-responsive chromatographic materials had been greatly developed by using various polymerized monomers or grafting different functional groups on the PNIPAAm chain. Temperature-responsive chromatographic materials can be classified into four categories according to the separation principle: Hydrophobic interaction chromatography [24,25,26], ion exchange chromatography [27,28], affinity chromatography [29,30], and size exclusion chromatography [31,32]. In the latest research, Japanese scientist Okano et al. synthesized many temperature-responsive materials [33,34], such as poly(NIPAAm-*co*-phenylalanine-OMe5), poly(NIPAAm-*co*-phenylalanine-OMe10), and poly(NIPAAm-*co*-tryptophan-OMe5) [35]. These three polymers were grafted onto aminopropyl silica by using an activated ester–amine coupling method, which exhibited a reversible hydrophilic/hydrophobic phase transition at their LCSTs. Temperature-responsive chromatography materials only use water as the mobile phase to separate sex steroids and phenylthiohydantoin-amino acids.

In addition, Liu et al. [36] used poly(NIPAAm-*co*-2-diethylaminoethyl methacrylate-*co*-*N*-*tert*-butylacrylamide)-grafted silica materials synthesized by atom transfer radical polymerization (ATRP) [37,38,39] to capture acidic proteins in human serum samples and increased the number of identified proteins that were detected. Nagase et al. [27,40] used poly(NIPAAm-*co*-acrylic acid-*co*-*N*-*tert*-butylacrylamide)-grafted silica materials synthesized by ATRP to capture and release catecholamine derivatives, angiotensin peptides, and angiotensin subtypes. 2-diethylaminoethyl methacrylate (DEAEMA) and acrylic acid (AAc) are alkaline and acidic functional groups, respectively, which are used to capture acidic and alkaline proteins in particular situations and enable the release of the proteins by changing the temperature only. Poly(NIPAAm-*co*-2-(dimethylamino)ethyl methacrylate-*co*-4-vinylphenylboronic acid)-grafted silica is an affinity chromatography stationary phase material, which contains the boronic acid functional group 4-vinylphenylboronic acid (VPBA) [41,42]. Compounds with *cis*-diols can be captured by boronic acid through the formation of five- or six-membered cyclic esters between the *cis*-diols and the boronic acid ligands [43]. The nitrogen atom in the 2-(dimethylamino)ethyl methacrylate (DMAEMA) structure can enhance the capture capacity of VPBA based on the principle of boronate affinity solid phase extraction [44]. Adenosine and glycoproteins containing dihydroxy groups have been captured and released using poly(NIPAAm-*co*-DMAEMA-*co*-VPBA)-grafted silica materials synthesized by atom transfer radical polymerization (ATRP). In the present study, reversible addition–fragmentation chain transfer (RAFT) [45,46,47] was used for the synthesis of temperature-responsive polymers. Compared to ATRP, the RAFT method could be carried out in a wide variety of solvents under mild conditions using different monomers, which has a wide range of applications.

Such temperature-responsive polymers are widely used in biomedical applications [48,49,50,51,52]. Steroids, amino acids, peptides, proteins, and other compounds can be separated using temperature-responsive materials as the stationary phase. Temperature-responsive chromatography avoids the use of organic solvents and has obvious advantages: It reduces environmental pollution, maintains the biological activity of the sample to the greatest extent and reduces the economic cost. Therefore, temperature-sensitive chromatography is a practical green chromatogram, making it more valuable in the biomedical field than traditional chromatography.

The separation of acidic proteins, alkaline proteins, and glycoproteins cannot be achieved using only one temperature-responsive chromatographic material. Therefore, in this study, we developed a method using the combination of three functionalized temperature-responsive chromatographic stationary phases to capture acidic proteins, alkaline proteins, and glycoproteins in mouse serum. These three modified, functionalized temperature-responsive chromatographic materials were synthesized by RAFT. Optimized grafting conformations enhanced the temperature-sensitivity, which was evaluated by the elution of steroids. The selective capture and release properties of the materials, which was affected by changing the temperature, were investigated with three standard proteins. Furthermore, the three materials were used to analyze the protein in mouse serum. Without switching the mobile phase, the capture and separation of proteins in mouse serum was achieved by the combination of three temperature-responsive chromatographic stationary phase materials. To our knowledge, this method is the first combinatorial use of three functionalized temperature-responsive chromatographic stationary phase silica materials to separate proteins in mouse serum. We believe that this combined method will be applied to the separation analysis of more types of biological samples in the future.

## 2. Results and Discussion

### 2.1. Preparation and Characterization of Chromatographic Materials

In our previous study [36,42], ATRP was used as the polymerization method. However, the metal ions used in ATRP, such as Cu^+^, are easily oxidized and it was difficult to completely remove the metal ions, which could influence the biological samples. Compared to ATRP, the RAFT method can be carried out in a wider variety of solvents. The reaction condition was mild and controllable, and the monomers had a wider range of applications. Therefore, we optimized the synthetic method to achieve the temperature-responsive polymers by the RAFT method.

**Figure 1:**
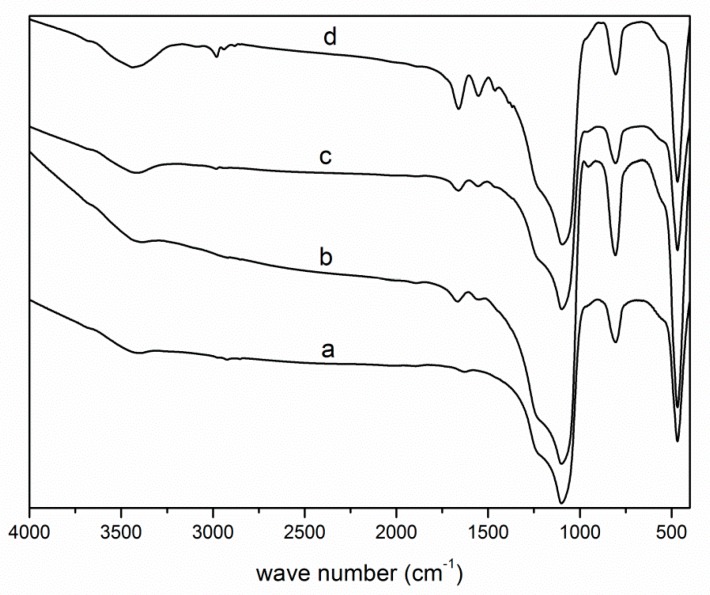
FTIR spectra of different silica in the synthesis of poly(NIPAAm-*co*-DEAEMA-*co*-*t*BAAm). a. NH_2_-grafted silica; b. CTA-grafted silica; c. poly(NIPAAm-*co*-DEAEMA-*co*-*t*BAAm)-grafted silica, the grafting density is 30%; d. poly(NIPAAm-*co*-DEAEMA-*co*-*t*BAAm)-grafted silica, the grafting density is 50%.

Three chromatographic materials were characterized by Fourier-transform infrared (FTIR) spectroscopy. The FTIR spectra of NH_2_-grafted silica, S-1-dodecyl-S′-(α,α′-dimethyl-α″-acetic acid)trithiocarbonate-grafted silica (chain transfer agent (CTA)-grafted silica), and poly(NIPAAm-*co*-DEAEMA-*co*-*t*BAAm)-grafted silica with different grafting densities are shown in Figure 1. The characteristic adsorption band from the bending vibration of N–H was observed at 1553 cm^−1^ and the stretching vibration of C=O was observed at 1665 cm^−1^, suggesting that the CTA was successfully grafted onto the silica surface. Compared to the CTA-grafted silica, C–H stretching vibrations at 2980 cm^−1^ and N–H stretching vibrations at 3440 cm^−1^ appeared. The spectrum showed stretching could strengthen with the increased grafting ratio. The results showed that the poly(NIPAAm-*co*-DEAEMA-*co*-*t*BAAm) was successfully grafted onto the silica surface. The FTIR spectroscopy of NH_2_-grafted silica, CTA-grafted silica, poly(NIPAAm-*co*-AAc-*co*-*t*BAAm)-grafted silica and poly(NIPAAm-*co*-DMAEMA-*co*-VPBA)-grafted silica with different grafting density are shown in Figures S1 and S2. The results showed that poly(NIPAAm-*co*-AAc-*co*-*t*BAAm) and poly(NIPAAm-*co*-DMAEMA-*co*-VPBA) were successfully grafted onto the silica surface.

To further confirm the successful synthesis of the three chromatographic materials, X-ray photoelectron spectroscopy (XPS) was applied to analyze the element constitution. The XPS spectra of NH_2_-grafted silica, CTA-grafted silica, and poly(NIPAAm-*co*-DEAEMA-*co*-*t*BAAm)-grafted silica with different grafting densities are shown in Table 1. The ratio of element S was increased from 0% to 1.44% and that of element N was increased from 1.45% to 2.37%, suggesting that the CTA was successfully grafted onto the silica surface. Compared to CTA-grafted silica, the ratio of element C was increased, and that of S and O were reduced. The change showed that the grafting ratio increased with the increasing grafting density of temperature-responsive chromatographic materials. The results showed that the poly(NIPAAm-*co*-DEAEMA-*co*-tBAAm) was successfully grafted onto the silica surface. The XPS spectra of NH_2_-grafted silica, CTA-grafted silica, poly(NIPAAm-*co*-AAc-*co*-tBAAm)-grafted silica, and poly(NIPAAm-*co*-DMAEMA-*co*-VPBA)-grafted silica with different grafting densities are shown as Table S1. The results showed that the poly(NIPAAm-*co*-AAc-*co*-tBAAm) and poly(NIPAAm-*co*-DMAEMA-*co*-VPBA) were successfully grafted onto the silica surface.

**Figure 2:**
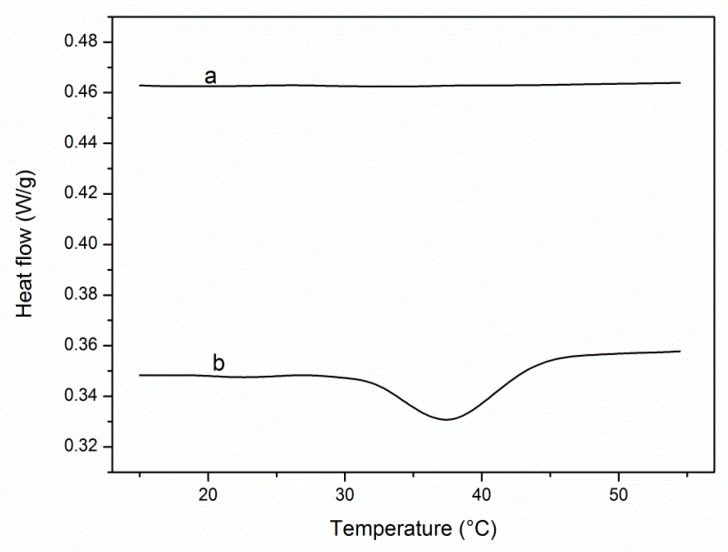
Drilling string compensator curves of different grafted silica. a. NH_2_-grafted silica, b. poly(NIPAAm-*co*-DEAEMA-*co*-*t*BAAm)-grafted silica, the grafting density is 30%.

A drilling string compensator (DSC) was applied to analyze the temperature-sensitivity of the material. High temperatures are necessary to release the proteins if the lower critical solution temperature (LCST) is too high, in which case, proteins will be destroyed under high temperature. Therefore, the designed three materials should have an appropriate LCST, which is close to 37 °C [53]. The DSC curves of NH_2_-grafted silica and poly(NIPAAm-*co*-DEAEMA-*co*-*t*BAAm)-grafted silica are shown in Figure 2. The results showed that the LCST of poly(NIPAAm-*co*-DEAEMA-*co*-*t*BAAm)-grafted silica was 37.7 °C. The LCSTs of poly(NIPAAm-*co*-AAc-*co*-tBAAm)-grafted silica and poly(NIPAAm-*co*-DMAEMA-*co*-VPBA) -grafted silica were 36.6 (Figure S3) and 35.80 °C (Figure S4), respectively. These LCSTs are close to the normal human physiological temperature, which is very important to avoid protein breakdown. The captured proteins were expected to be released at a temperature less than 50 °C, which is suitable for proteins.

**Figure 3:**
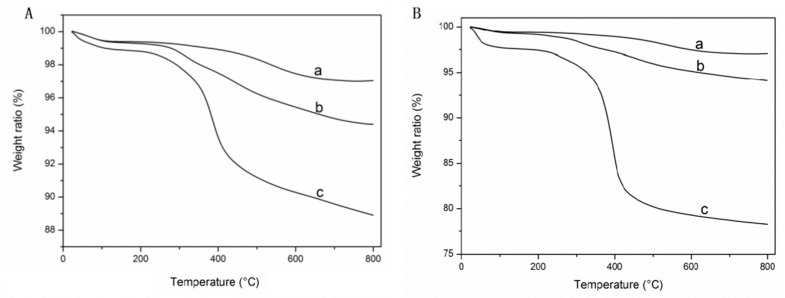
Thermogravimetric analysis curves of different grafted silica. a. NH_2_-grafted silica; b. CTA-grafted silica; c. poly(NIPAAm-*co*-DEAEMA-*co*-tBAAm)-grafted silica. (**A**) The grafting density is 30%; (**B**) the grafting density is 50%.

Based on our previous study, the grafting ratio and the density of the polymers on the modified silica determine the capture and release properties directly [54]. Therefore, optimization of the grafting conformation is necessary. Grafting density is controlled by the modified CTA, and the grafting ratio of target materials can be determined by thermogravimetric analysis (TGA). The grafting ratio was calculated using the following equation:

(1) $$\mathrm{Graft}\;\mathrm{ratio}/\%=\frac{\Delta \mathrm{WSIN}-\Delta \mathrm{WSI}}{1-\Delta \mathrm{WSI}}\times 100\%,$$

where ΔWSI and ΔWSIN are the weight loss ratios of the CTA-modified silica and the polymer-modified silica, within the temperature range of 100–750 °C, respectively. The TGA curves of NH_2_-grafted silica, CTA-grafted silica, and poly(NIPAAm-*co*-DEAEMA-*co*-tBAAm)-grafted silica with different grafting density are shown in Figure 3. The grafting ratios of poly(NIPAAm-*co*-DEAEMA-*co*-*t*BAAm)-grafted silica 30% and poly(NIPAAm-*co*-DEAEMA-*co*-*t*BAAm)-grafted silica 50% were 4.86% and 14.16%, respectively. The poly(NIPAAm-*co*-AAc-*co*-*t*BAAm) 30%, poly(NIPAAm-*co*-AAc-*co*-*t*BAAm) 50%, and poly(NIPAAm-*co*-DMAEMA-*co*-VPBA) 50% were 6.83% (Figure S5), 14.45% (Figure S5), and 10.25% (Figure S6), respectively. The best grafting ratios range from 10% to 20% [55]. Therefore, poly(NIPAAm-*co*-DEAEMA-*co*-*t*BAAm) 50%, poly(NIPAAm-*co*-AAc-*co*-*t*BAAm) 50%, and poly(NIPAAm-*co*-DMAEMA-*co*-VPBA) 50% were used in the subsequent experiments to enrich steroids and proteins.

### 2.2. Temperature Sensitivity of the Materials

**Figure 4:**
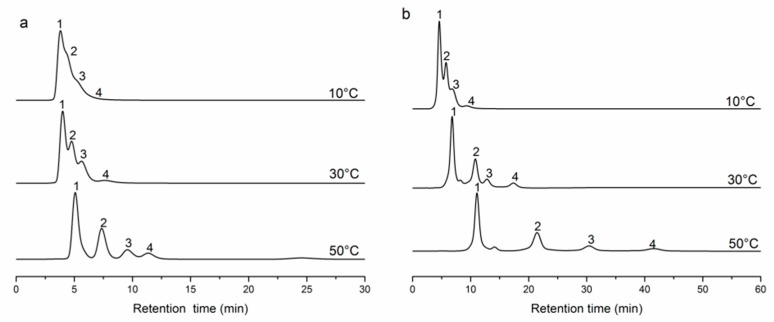
**Figure4.** Chromatograms of steroids separated on polymer-modified silica columns. (**a**) Poly(NIPAAm-*co*-DEAEMA-*co*-tBAAm)-grafted silica, grafting density is 30%; (**b**) poly(NIPAAm-*co*-DEAEMA-*co*-tBAAm)-grafted silica, grafting density is 50%. The mobile phase used was 10 mM pH 7 phosphate buffer solution; flow rate, 0.1 mL/min; detection wavelength, 254 nm. Peaks: 1, hydrocortisone; 2, dexamethasone; 3, hydrocortisone butyrate; 4, prednisolone acetate.

Four steroids were used to evaluate the temperature sensitivity and the separation performance of the silica materials at 10, 30, and 50 °C [56,57]. As shown in Figure 4, the retention time of the steroids and the number of peaks increased with increasing temperature. The properties of the materials were determined by the grafting density and ratio of the polymers directly. The separation performance of poly(NIPAAm-*co*-DEAEMA-*co*-*t*BAAm)-grafted silica with a 14.16% grafting ratio was better than that with a 4.86% grafting ratio (as shown in Figure 4a,b). The steroids have higher solubility in water at higher temperatures, which could lead to a shorter retention time because of the strong van der Waals forces with water. However, the hydrophobic interactions between the steroids and polymers are enhanced as the temperature increases, which result in longer retention times. The competition between the above two factors resulted in a longer retention time of the steroids at higher temperatures because the hydrophobic interaction was the prominent factor. This analysis of steroids indicated that the modified silica columns had excellent temperature-responsive properties. The poly(NIPAAm-*co*-AAc-*co*-tBAAm)-grafted silica and the poly(NIPAAm-*co*-DMAEMA-*co*-VPBA)-grafted silica had the similar properties (Figures S7 and S8).

### 2.3. Characterization of Capture Performance

**Figure 5:**
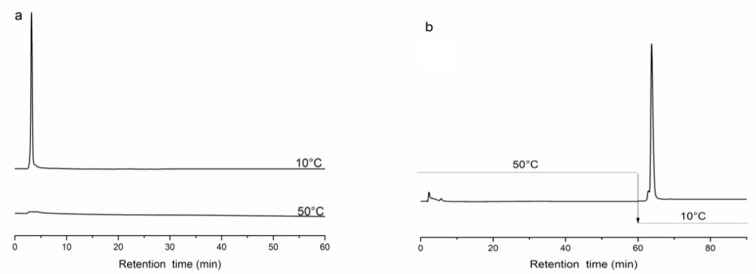
Chromatogram of bovine serum albumin (BSA) using a poly(NIPAAm-*co*-DEAEMA-*co*-tBAAm)-grafted silica column. (**a**) Chromatogram of BSA at 10 and 50 °C; (**b**) injecting the sample at 50 °C and changing the temperature to 10 °C at 60 min, flow rate 0.1 mL/min, detection wavelength 280 nm, sample size 5 μL.

Bovine serum albumin (BSA) was used as the acidic protein to examine the capture and release properties of poly(NIPAAm-*co*-DEAEMA-*co*-*t*BAAm)-grafted silica [36]. Figure 5 shows that the copolymer with alkaline groups could strongly capture the acidic protein at 50 °C and release the protein within 5 min at 10 °C. These results indicated that the selective capture and release of proteins could be achieved by modulating the surface properties of the chromatographic material. The capture and rapid-release ability depended on a substantial and fast change in the charges of the grafted copolymers. The separation of acidic proteins could be achieved by simply adjusting the charge of the copolymers with a temperature increase [36]. At 50 °C, the copolymer exhibited hydrophobic properties and the polymer chains collapsed and, because of strong interactions between the proteins and copolymers, the acidic target proteins were captured. The copolymer chains became hydrophilic and expanded as the temperature changed from 50 to 10 °C. Changes in the conformation and hydrophobicity of the copolymers weakened the interactions between the captured acidic target proteins and the copolymer. These changes resulted in the captured acidic target proteins being quickly released.

**Figure 6:**
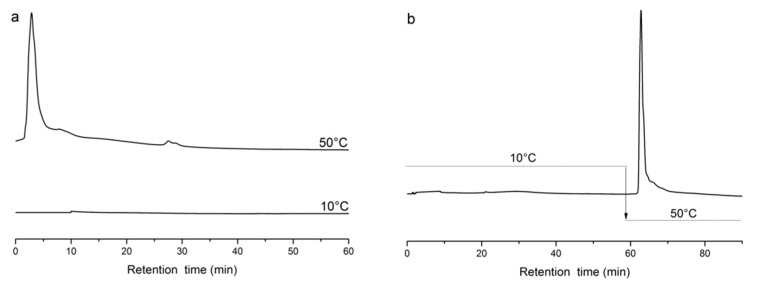
Chromatograms of protamine using a poly(NIPAAm-*co*-AAc-*co*-tBAAm)-grafted silica column. (**a**) Chromatogram of protamine at 10 and 50 °C; (**b**) injecting the sample at 10 °C and increasing temperature to 50 °C at 60 min, flow rate 0.1 mL/min, detection wavelength 280 nm, sample size 5 μL.

Protamine was used as an alkaline protein to examine the capture and release properties of poly(NIPAAm-*co*-AAc-*co*-*t*BAAm)-grafted silica [58]. Figure 6 shows that the alkaline target protein was captured and released by the copolymer with acidic groups [40]. At 10 °C, the alkaline protein was captured by the stationary phase when the polymer chains were stretched through electrostatic interactions. The polymer chains were in the form of collapsed globules at 50 °C, and the acidic functional groups were covered by the chains. The alkaline target protein was quickly released through hydrophobic interactions.

**Figure 7:**
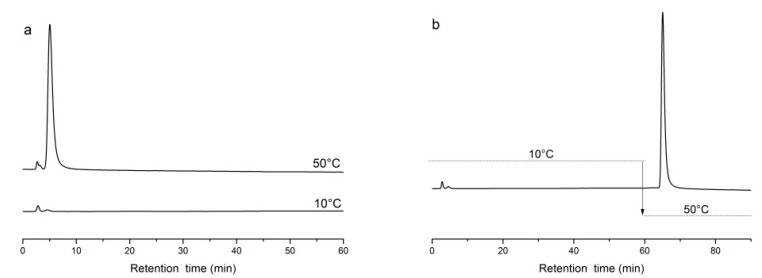
Chromatograms of adenosine using a poly(NIPAAm-*co*-DMAEMA-*co*-VPBA)-grafted silica column. (**a**) Chromatogram of adenosine at 10 and 50 °C; (**b**) injecting the sample at 10 °C and changing the temperature to 50 °C at 60 min, flow rate 0.1 mL/min, detection wavelength 280 nm, sample size 5 μL.

To evaluate the capture and release properties of poly(NIPAAm-*co*-DMAEMA-*co*-VPBA)-grafted silica, we used adenosine and horseradish peroxidase (HRP) as analytes [43,44]. The copolymer captured adenosine and HRP at 50 °C and released them at 10 °C (Figure 7). DMAEMA was strongly hydrophilic resulting in a reduction in the pH of the capture molecule [42,59]. Glycoproteins and nucleotides contain *cis*-diols and form tetrahedral structure with the boron atom of VPBA. At 10 °C, the copolymer chains were in the form of extended random coils. Proteins containing *cis*-diols can form cyclic esters with charged boronic acid molecules. At 50 °C, the copolymer chains were in the form of collapsed globules and the target proteins were quickly released.

### 2.4. Combination of Three Functional Temperature-Sensitive Chromatographic Materials for Serum Protein Analysis

**Figure 8:**
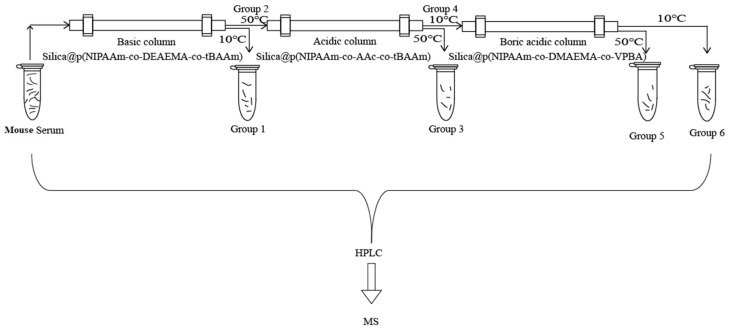
Workflow for the proteomics of mouse serum using three thermal-responsive silica materials.

Mouse serum is a typical complex biological sample that includes a variety of proteins. The removal of abundant proteins from complex biological samples has been demonstrated to vastly increase the number of individual proteins that can be identified in a mixture [60]. The proteomic analysis of mouse serum was used to evaluate the separation capability of the temperature-responsive silica materials in the pretreatment of complex biosamples. The three functionalized materials were able to capture acidic proteins, alkaline proteins, and glycoproteins. Proteins could be rapidly released by just changing the temperature as indicated by the results described above. The workflow for the treatment of the mouse serum is shown in Figure 8.

**Figure 9:**
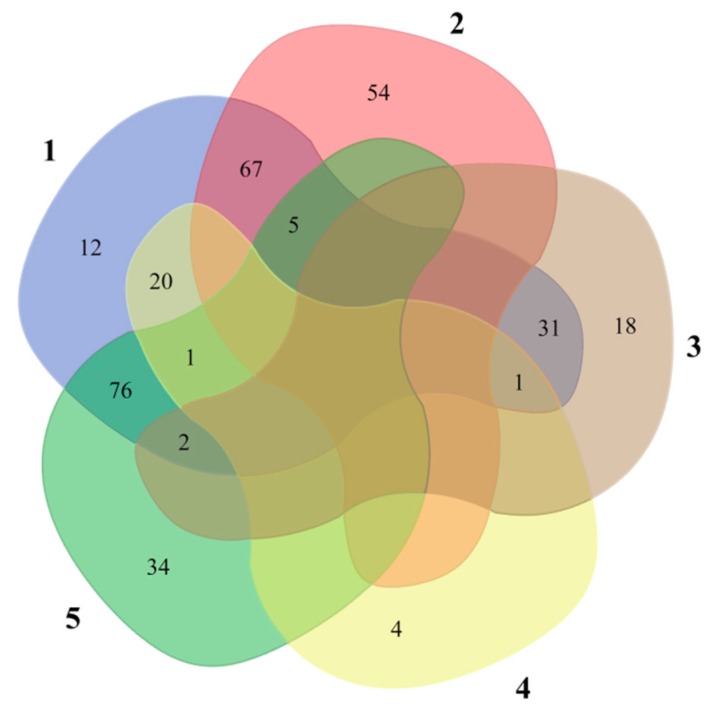
Proteomic analysis of mouse serum. Part 1: Diluted mouse serum; Part 2: Mouse serum treated with poly(NIPAAm-*co*-DEAEMA-*co*-tBAAm)-grafted silica column at 10 °C (Group 1); Part 3: Serum treated with poly(NIPAAm-*co*-AAc-*co*-*t*BAAm)-grafted silica column at 50 °C (Group 3); Part 4: Mouse serum treated with poly(NIPAAm-*co*-DMAEMA-*co*-VPBA)-grafted silica column at 50 °C (Group 5); Part 5: Mouse serum treated with poly(NIPAAm-*co*-DMAEMA-*co*-VPBA)-grafted silica column at 10 °C (Group 6).

First, the crude mouse serum was directly analyzed by MS/MS. The obtained protein results are shown in Table S2. Only 215 proteins were identified without the capture/release pretreatment. The poly(NIPAAm-*co*-DEAEMA-*co*-*t*BAAm)-grafted silica column was used for proteomic analysis of the crude mouse serum. Group 1 was collected by temperature-modulated capture at 50 °C and release at 10 °C. The obtained protein results are shown in Table S3. A total of 126 acidic proteins were identified in Group 1. Compared with the crude mouse serum, there were 54 different acidic proteins found in Group 1. Then, the poly(NIPAAm-*co*-AAc-*co*-*t*BAAm)-grafted silica column was used for proteomic analysis of Group 2. Group 3 was collected by temperature-modulated capture at 10 °C and release at 50 °C. The obtained protein results are shown in Table S4. A total of 52 alkaline proteins were identified in Group 3. Compared to the crude mouse serum, 18 alkaline proteins were found in Group 3. Finally, the poly(NIPAAm-*co*-DMAEMA-*co*-VPBA)-grafted silica column was used for proteomic analysis of Groups 5 and 6. Group 5 was collected by temperature-modulated capture at 10 °C and release at 50 °C. The obtained protein results are shown in Table S5. A total of 26 glycoproteins were identified in Group 5. Compared with the crude mouse serum, four glycoproteins were found in Group 5. Group 6 was collected by temperature-modulated release at 10 °C. As shown in Table S6, 118 proteins were identified in Group 6. Thirty-four proteins in Group 6 were different from those identified in the crude mouse serum. Therefore, 313 proteins were identified from the mouse serum sample with the capture/release pretreatment, while only 215 proteins were identified without the capture/release pretreatment (Figure 9). The number of different proteins obtained by the temperature-controlled capture and release strategy was 1.46 times that found using the crude mouse serum. As shown in Figure 9, we observed that five acidic proteins were not completely captured by the poly(NIPAAm-*co*-DEAEMA-*co*-*t*BAAm)-grafted silica column, three alkaline proteins were not completely captured by the poly(NIPAAm-*co*-AAc-*co*-*t*BAAm)-grafted silica column, and one glycoprotein was not completely captured by the poly(NIPAAm-*co*-DMAEMA-*co*-VPBA)-grafted silica column. Steric hindrance might be the reason for this failure to completely capture these proteins. As discussed above, the chain conformations and interface properties of the three functionalized thermally switchable materials could be altered to enable the rapid release of proteins under thermal stimuli. The capture and release of the target proteins was simply carried out by changing the temperature of the temperature-responsive polymer-modified silica column, without changing the composition of the mobile phase to a solvent that is not compatible with MS analysis. This approach eliminates the time-consuming need for using organic solvents or the buffer-changing steps in LC MS/MS analysis. These results showed the advantages of using functionalized temperature-responsive chromatographic materials in the pretreatment of proteins from complex biological samples.

## 3. Materials and Methods

### 3.1. Materials and Instruments

BSA, HRP, and protamine were purchased from Sigma-Aldrich. NIPAAm, DEAEMA, and DMAEMA were obtained from TCI (Shanghai, China). AAC, VPBA, *t*BAAm, azodiisobutyronitrile (AIBN), 4-dimethylaminopyridine (DMAP), *N*,*N*’-diisopropylcarbodiimide (DIC), adenosine, and dopamine were obtained from J and K Chemical (Beijing, China). 3-Aminopropyl silica (silica@NH_2_, diameter, 5 μm; pore size, 100 Å) was purchased from Boinna-Agela Technologies (Tianjin, China). Hydrocortisone, prednisolone acetate, dexamethasone, and hydrocortisone butyrate were purchased from the National Institute for Food and Drug Control (Beijing, China). All the chemicals were analytical grade. DEAEMA and DMAEMA were purified by an Al_2_O_3_ column to remove *p*-hydroxyanisole (MEHQ) before use.

FTIR spectra were obtained using a PerkinElmer (Boston, MA, USA) FTIR spectrometer. HPLC spectra were obtained using a Shimadzu LC-20AT HPLC system with a Shimadzu SPD-20A UV detector. The temperature was controlled by a CTO-20AC column controller (Shimadzu, Japan). Elemental analysis was conducted using an Elemental Vario MICRO CUBE analyzer (Germany). TGA results were obtained using a Discovery TGA instrument (TA Instruments, USA). Differential scanning calorimetry (DSC) spectra were obtained using a Q2000 DSC instrument (TA Instruments, USA).

### 3.2. Synthesis of CTA-Grafted Silica

3-Aminopropyl silica (1.5 g), DMAP (0.6 g, 4.9 mmol), CTA (0.486 g, 1.34 mmol), and *N*-acetylglycine (0.809 g, 0.55 mmol) were mixed in dichloromethane (60 mL) in a round-bottom flask with three necks. The grafting density of the polymers was controlled by the molar ratio of CTA to DMAP. The reaction mixture was cooled to 10 °C under nitrogen. After vacuuming and purging the reaction mixture with nitrogen three times, DIC (6 mL) was then added over 30 min. The reaction was stirred for 48 h at room temperature and then exposed to air to stop the reaction. The products were washed with dichloromethane, methanol, and ethanol three times. CTA-grafted silica was then produced after drying for 8 h at 60 °C.

CTA [55] was synthesized according to a previous study and was characterized by FTIR and ^1^H NMR (shown in Figures S9 and S10).

### 3.3. Synthesis and Characterization of the Three Temperature-Responsive Materials

The synthesis of poly(NIPAAm-*co*-DEAEMA-*co*-*t*BAAm)-grafted silica was performed by mixing. CTA-grafted silica (0.4 g), NIPAAm (1.732 g, 15.32 mmol), DEAEMA (155 μL, 0.77 mmol), and *t*BAAm (0.195 g, 1.53 mmol) were mixed in a round-bottom flask with three necks. The reaction mixture was cooled to 10 °C under nitrogen. After vacuuming and purging the reaction mixture with nitrogen three times, AIBN (14.5 mg, 0.088 mmol) was then added. The reaction was then stirred for 12 h at 70 °C and then exposed to air to stop the reaction. The products were washed with methanol, ethanol, and deionized water three times. Poly(NIPAAm-*co*-DEAEMA-*co*-*t*BAAm)-grafted silica was finally produced after drying for 8 h at 60 °C.

CTA-grafted silica (0.4 g), NIPAAm (1.732 g, 15.32 mmol), AAc (53 μL, 0.77 mmol), and *t*BAAm (0.195 g, 1.53 mmol) were mixed in a round-bottom flask with three necks. The reaction mixture was cooled to 10 °C under nitrogen. After vacuuming and purging the reaction mixture with nitrogen three times, AIBN (14.5 mg, 0.088 mmol) was then added. The reaction was then stirred for 12 h at 70 °C and then exposed to air to stop the reaction. The products were washed with methanol, ethanol, and deionized water three times. Poly(NIPAAm-*co*-AAc-*co*-*t*BAAm)-grafted silica was finally produced after drying for 8 h at 60 °C.

CTA-grafted silica (0.4 g), NIPAAm (1.732 g, 15.32 mmol), DMAEMA (260 μL, 1.53 mmol), and VPBA (0.226 g, 1.53 mmol) were mixed in a round-bottom flask with three necks. The reaction mixture was cooled to 10 °C under nitrogen. After vacuuming and purging the reaction mixture with nitrogen three times, AIBN (14.5 mg, 0.088 mmol) was then added. The reaction was stirred for 12 h at 70 °C and then exposed to air to stop the reaction. The products were washed with methanol, ethanol, and deionized water three times. The poly(NIPAAm-*co*-DMAEMA-*co*-VPBA)-grafted silica was then dried for 8 h at 60 °C.

**Figure 10:**
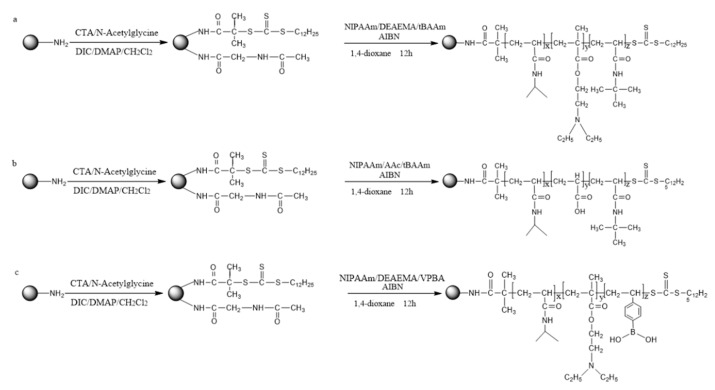
Synthesis of temperature-responsive polymer modified silica. (**a**) Synthesis of poly(NIPAAm-*co*-DEAEMA-*co*-*t*BAAm)-grafted silica; (**b**) synthesis of poly(NIPAAm-*co*-AAc-*co*-tBAAm)-grafted silica; and (**c**) synthesis of poly(NIPAAm-*co*-DMAEMA-*co*-VPBA)-grafted silica.

The synthetic routes for the functionalized temperature-responsive materials are shown in Figure 10. TGA was performed at a heating rate of 10 °C/min from ambient temperature to 800 °C under a nitrogen atmosphere to determine the grafting amount of the copolymers on the silica surfaces. The three functionalized temperature-responsive chromatographic materials were characterized by FTIR, elemental analysis, and DSC.

### 3.4. Chromatographic Assay for the Separation and Enrichment of Steroids and Proteins

The packing method used was the methanol homogenate filling method [61,62]. Silica materials (0.318 g), synthesized as described above, were pushed into a stainless-steel column (50 mm × 2.1 mm i.d.) with methanol as the solvent under a maximum pressure of 50 MPa. Hydrocortisone (4 mg), dexamethasone (4 mg), prednisolone acetate (4 mg), and hydrocortisone butyrate (4 mg) were dissolved in ethanol (3 mL). Then, water (20 mL) was added to yield a concentration of 0.174 mg/mL. Phosphate buffer (10 mM) at pH 7.0 was used for the temperature-responsive elution of the steroids with a flow rate of 0.1 mL/min and UV monitoring at a wavelength of 254 nm. Solutions of protein samples were prepared at a concentration of 1 mg/mL for standard solutions. Protein capture and release were monitored at a wavelength of 280 nm with a flow rate of 0.1 mL/min.

### 3.5. Combination of Three Functional Temperature-Sensitive Chromatographic Materials

The supernatant was obtained from fresh mouse blood centrifuged at 3000 rpm for 10 min at room temperature. Mouse serum was diluted 20 times before use. The workflow for the treatment of the mouse serum is shown in Figure 8. First, the samples were enriched using a poly(NIPAAm-*co*- DEAEMA-*co*-*t*BAAm) column for 30 min at 50 °C (Figure S11) with release at 10 °C. Group 1 was the collected elution peak at 10 °C, and Group 2 was the collected breakthrough peak from the basic column at 50 °C. Group 2 was enriched for 30 min at 10 °C using a poly(NIPAAm-*co*-AAc-*co*-*t*BAAm) column (Figure S12). Then, the collected components were denoted as Group 4, and the liquid that flowed from the column when the temperature was increased to 50 °C was denoted as Group 3. Finally, Group 4 was enriched using a poly(NIPAAm-*co*-DMAEMA-*co*-VPBA) column, Group 5 was the collected elution peak at 10 °C (Figure S13), and Group 6 was the collected breakthrough peak at 50 °C. The obtained proteins were analyzed by LC/MS/MS [36].

The protein samples were denatured and reduced using a solution of 10 mM DTT, 50 mM NH_4_HCO_3_, and 8 M urea for 4 h at 37 °C. The solutions were alkylated with 50 mM iodoacetamide at room temperature for 1 h in the dark, and then diluted to 1 M urea with 50 mM NH_4_HCO_3_. Trypsin was added at a concentration of 50:1 and the mixture was incubated for 20 h at 37 °C in a water bath. The digested samples were lyophilized and then desalted by C18 solid phase extraction (SPE) cartridges. The peptides were first separated by strong cation exchange chromatography at 0.2 mL/min with an Agilent 1100 series device. Mobile phase A was composed of 10 mM ammonium formate and 25% acetonitrile at pH 3.0. Mobile phase B was composed of 500 mM ammonium formate and 25% acetonitrile at pH 6.8. Gradient elution was performed under 0% mobile phase B isocratic conditions for 10 min, followed by a gradient from 0% to 50% of mobile phase B over 50 min, a gradient from 50% to 100% of mobile phase B over 10 min, and 100% mobile phase B for an additional 10 min. Fractions were collected every 2 min and lyophilized.

The peptides were identified by MS using an Agilent HPLC-Chip/Q-TOF MS system. The HPLC-Chip configuration had a 160 nL online enrichment column and an analytical column. Mobile phase A was composed of 0.1% formic acid in water. Mobile phase B was composed of 0.1% formic acid in acetonitrile. The gradient method was used for HPLC separation with a gradient from 3% to 40% of mobile phase B over 60 min, followed by 40% to 95% of mobile phase B over 5 min, an isocratic condition of 95% mobile phase B for 10 min, and from 95% to 3% of mobile phase B over 5 min. The column was equilibrated for 5 min before each use. The flow rate was 4 μL/min and the samples were eluted at 400 nL/min. In the positive ion mode, MS analysis was performed on a quadrupole-time of flight (Q-TOF) MS system with an electrospray ionization ESI source. The MS parameters were as follows: A gas temperature of 300 °C, the skimmer was set at 65 V, the capillary voltage was set at 1700 V, the octopole radio frequency voltage was set at 750 V, the fragmentor was set at 175 V, and the drying gas flow was set at 3 L/min. G2721AA Spectrum Mill software was used for analyzing MS/MS data against the Swiss-Prot database.

## 4. Conclusions

In this study, we developed an approach in which a combination of three functionalized temperature-responsive chromatographic stationary phases was able to easily capture and release acidic proteins, alkaline proteins, and glycoproteins in the pretreatment of mouse serum. Steroids and proteins were separated by changing the temperature, and the method showed an outstanding temperature-responsive separation performance. These three functionalized materials were also used in the pretreatment of mouse serum, with 313 proteins successfully identified. The number of different proteins identified was 1.46 times greater than the number identified without using the functionalized temperature-sensitive materials. The separation of temperature-responsive chromatogram materials was achieved by controlling the temperature to change the surface properties of the stationary phase and increasing the selectivity of the column using the same mobile phase. We believe that this combined approach will be applied to the separation process to help with the analysis of nucleic acid, proteomes, and other biological samples.

## Figures and Tables

**Table 1:** The XPS results of NH_2_-grafted silica, CTA-grafted silica, and poly(NIPAAm-*co*-DEAEMA-*co*-*t*BAAm)-grafted silica.

Element	C1s (%)	O1s (%)	N1s (%)	S2p (%)
NH_2_-grafted silica	39.61	58.94	1.45	0
CTA-grafted silica 30%	49.47	47.16	1.67	1.71
CTA-grafted silica 50%	46.50	49.68	2.37	1.44
poly(NIPAAm-*co*-DEAEMA-*co*-*t*BAAm)-grafted silica 30%	53.65	41.95	3.21	1.19
poly(NIPAAm-*co*-DEAEMA-*co*-*t*BAAm)-grafted silica 50%	75.18	21.97	2.04	0.81

## Supplementary Materials

The following are available online at https://www.mdpi.com/1420-3049/24/14/2626/s1, Figure S1. FTIR spectrum of different silica in the synthesis of poly(NIPAAm-*co*-AAc-*co*-*t*BAAm). Figure S2. FTIR spectrum of different silica in the synthesis of poly(NIPAAm-*co*-DMAEMA-*co*-VPBA). Figure S3. DSC curves of different grafted silica. Figure S4. DSC curves of different grafted silica. Figure S5. TGA curves of different grafted silica. Figure S6. TGA curves of different grafted silica. Figure S7. Chromatograms of steroids separated on polymer-modified silica columns. Figure S8. Chromatograms of steroids separated on polymer-modified silica column. Figure S9. FTIR spectrum of the CTA. Figure S10. ^1^H NMR spectrum of CTA. Figure S11. Chromatogram of mouse serum treated with poly(NIPAAm-*co*-DEAEMA-*co*-tBAAm)-grafted silica at 50 °C. Figure S12. Chromatogram of Group 2 treated with poly(NIPAAm-*co*-AAc-*co*-tBAAm)-grafted silica at 10 °C. Figure S13. Chromatogram of Group 4 treated with poly(NIPAAm-*co*-DMAEMA-*co*-VPBA)-grafted silica at 10 °C. Table S1. The XPS results of NH_2_-grafted silica, CTA-grafted silica, poly(NIPAAm-*co*-AAc-*co*-tBAAm)-grafted silica, and poly(NIPAAm-*co*-DMAEMA-*co*-VPBA)-grafted silica. Table S2. Type of proteins in crude mouse serum. Table S3. Type of proteins in group 1. Table S4. Type of proteins in Group 3. Table S5. Type of proteins in Group 5. Table S6. Type of proteins in Group 6.
