# Supplementary material for: Combination of Three Functionalized Temperature-Sensitive Chromatographic Materials for Serum Protein Analysis
Source: Molecules. 2019 Jul 19;24(14):2626. doi: 10.3390/molecules24142626 (PMC6680567; doi:10.3390/molecules24142626)
Supplement: Supplementary file 1 [file molecules-24-02626-s001.pdf]

Combination of Three Functionalized Temperature-Sensitive Chromatographic Materials for Serum Protein Analysis

Weiwei Sun[1], Rongji Dai[1], Bo Li[1,2], Guoxin Dai[1], Di Wang[1], Dandan Yang[1], Pingping Chu[1], Yulin Deng[1,*] and Aiqin Luo[1,*]

[1] School of Life Science, Beijing Institute of Technology, Beijing 100081, China; sunweiwei0411@163.com (W.S.); dairongji@126.com (R.D.); boli@bit.edu.cn (B.L.); 2120171424@bit.edu.cn (G.D.); wangdi19920719@163.com (D.W.); alicey_0724@163.com (D.Y.); chuhbxg@gmail.com (P.C.)

[2] Advanced Research Institute of Multidisciplinary Science, Beijing Institute of Technology, China

* Correspondence: deng@bit.edu.cn (Y.D.); aqluobit@163.com (A.L.); Tel: +86-13501125130 (Y.D.); +86-13901232393 (A.L.)

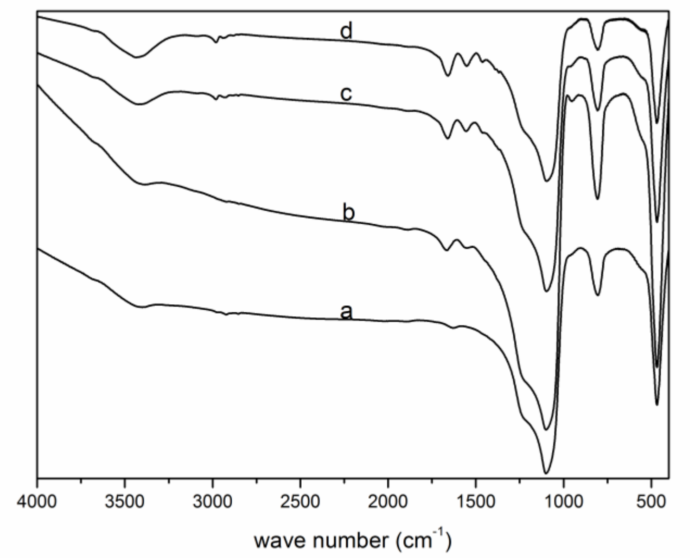

Figure S1. FTIR spectrum of different silicas in the synthesis of poly(NIPAAm-*co*-AAc-*co*-*t*BAAm). a. NH_2-grafted silica; b. CTA-grafted silica; c. poly(NIPAAm-*co*-AAc-*co*-*t*BAAm)-grafted silica, the grating density is 30%; d. poly(NIPAAm-*co*-AAc-*co*-*t*BAAm)-grafted silica, the grating density is 50%.

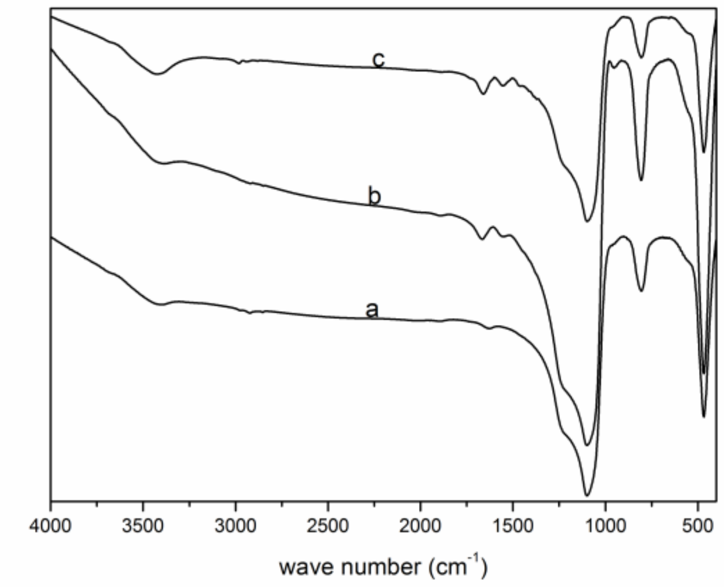

Figure S2. FTIR spectrum of different silicas in the synthesis of poly(NIPAAm-*co*-DMAEMA-*co*-VPBA). a. NH_2-grafted silica; b. CTA-grafted silica; c. poly(NIPAAm-*co*-DMAEMA-*co*-VPBA) -grafted silica, the grating density is 50%.

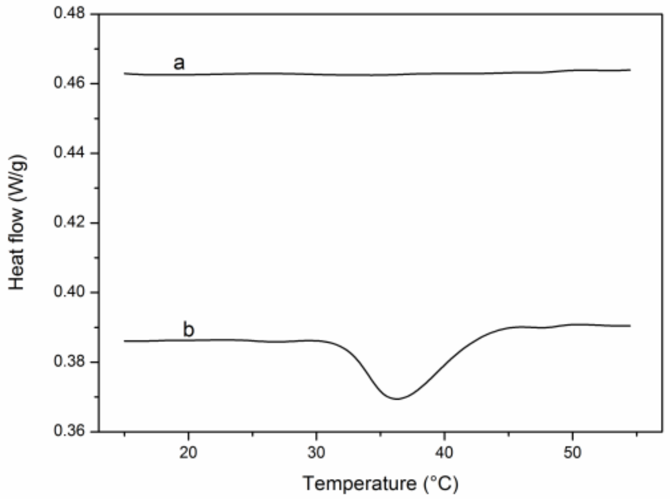

Figure S3. Drilling string compensator curves of different grafted silica. a. NH_2-grafted silica; b. poly(NIPAAm-*co*-AAc-*co*-*t*BAAm)-grafted silica, the grating density is 50%.

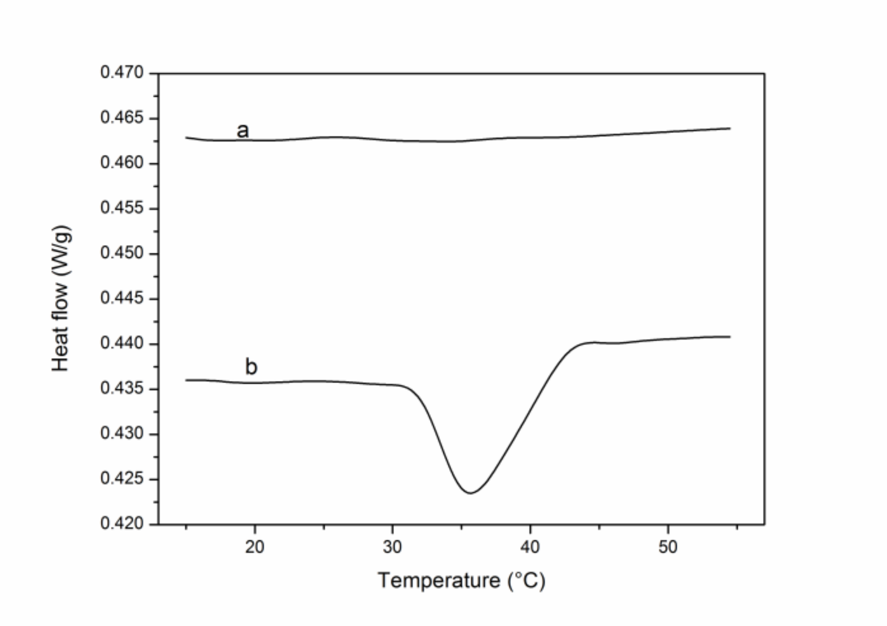

Figure S4. DSC curves Drilling string compensator curves of different grafted silica. a. NH_2-grafted silica; b. poly(NIPAAm-*co*-DMAEMA-*co*-VPBA)-grafted silica, the grating density is 50%.

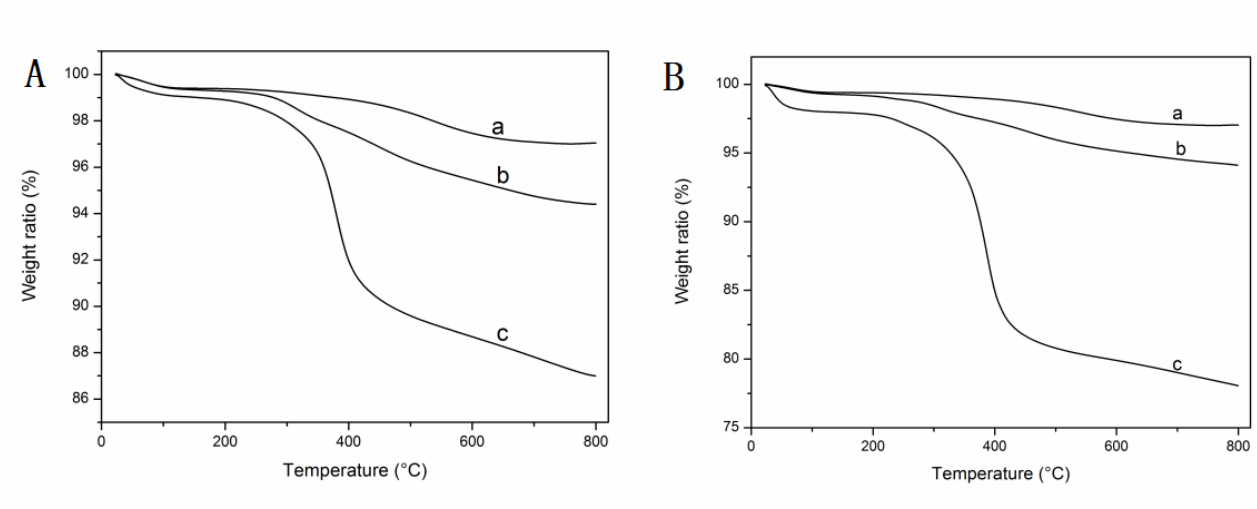

Figure S5. Thermogravimetric analysis curves of different grafted silica. a. NH_2-grafted silica; b. CTA-grafted silica; c. poly(NIPAAm-*co*-AAc-*co*-*t*BAAm)-grafted silica. (A) the grafting density is 30%; (B) the grafting density is 50%.

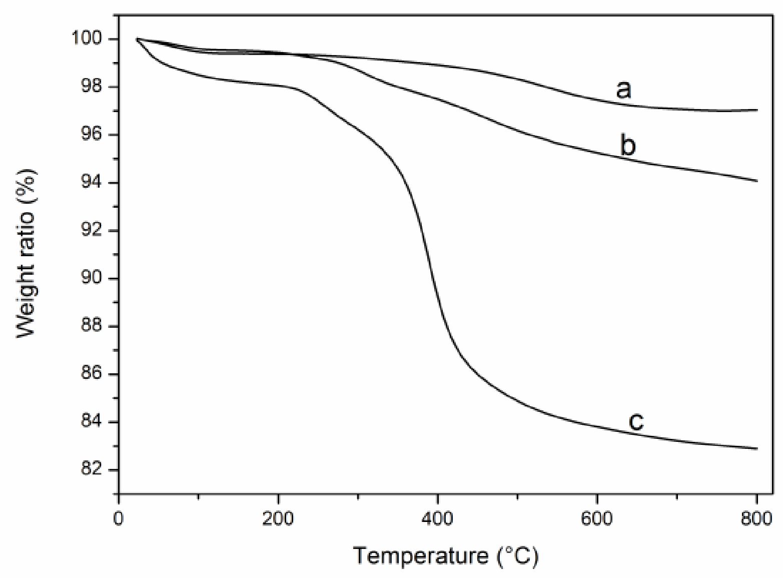

Figure S6. Thermogravimetric analysis curves of different grafted silica. a. NH_2-grafted silica; b. CTA-grafted silica; c. poly(NIPAAm-*co*-DMAEMA-*co*-VPBA)-grafted silica, the grating density is 50%.

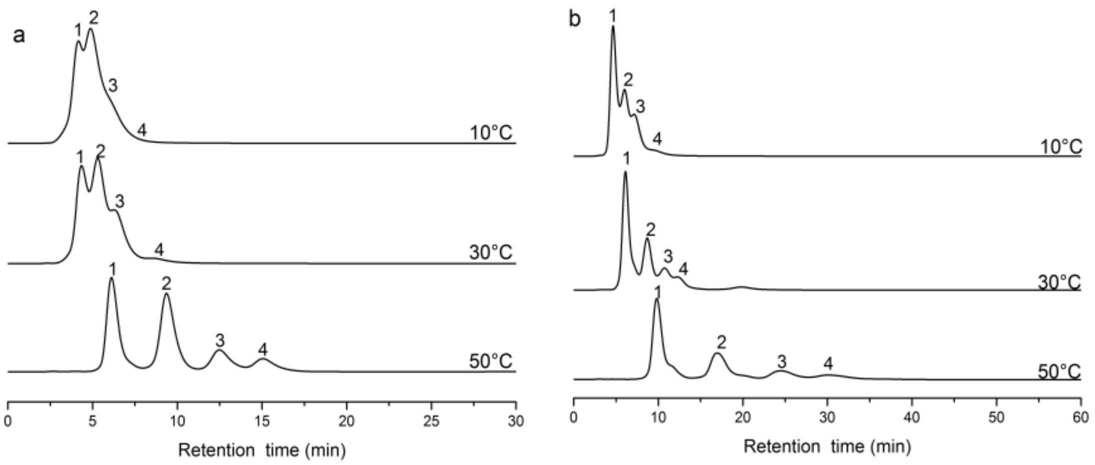

Figure S7. Chromatograms of steroids separated on polymer modified silica columns. (a) Poly(NIPAAm-*co*-AAc-*co*-*t*BAAm)-grafted silica, grafting density is 30%; (b) poly(NIPAAm-*co*-AAc-*co*-*t*BAAm)-grafted silica, grafting density is 50%. The mobile phase used was 10 mM pH 7 phosphate buffer solution; flow rate, 0.1 mL/min; detection wavelength, 254 nm. Peaks: 1, hydrocortisone; 2, dexamethasone; 3, hydrocortisone butyrate; 4, prednisolone acetate.

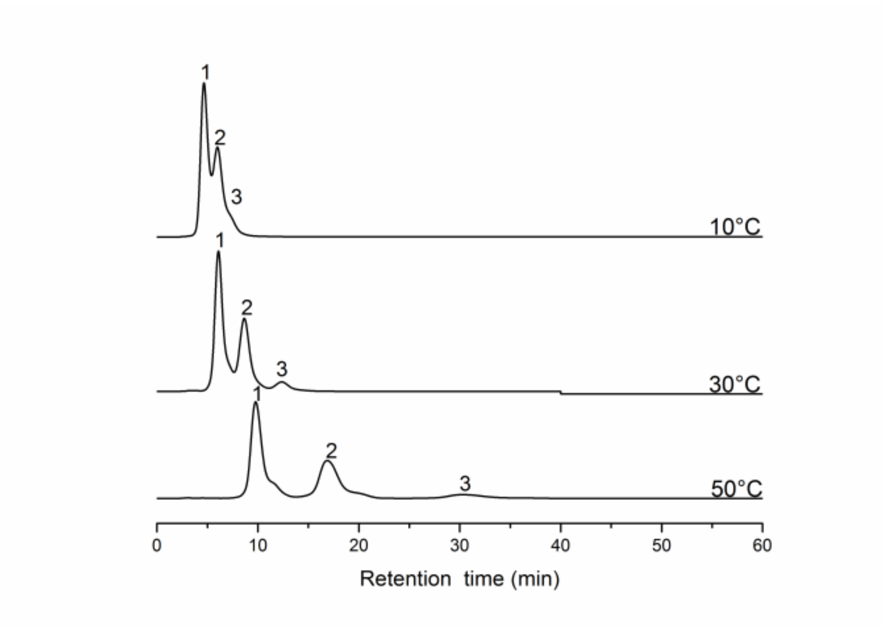

Figure S8. Chromatograms of steroids separated on polymer modified silica column. poly(NIPAAm-*co*-DMAEMA-*co*-VPBA)-grafted silica, grafting density is 50%. The mobile phase used was 10 mM pH 7 phosphate buffer solution; flow rate, 0.1 mL/min; detection wavelength, 254 nm. Peaks: 1, hydrocortisone; 2, dexamethasone; 3, hydrocortisone butyrate; 4, prednisolone acetate.

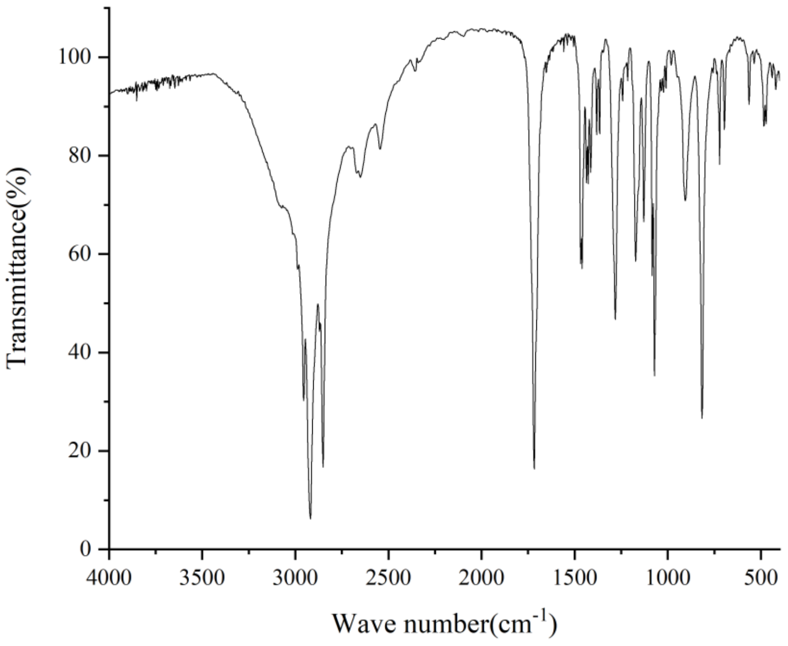

Figure S9. FTIR spectrum of the CTA.

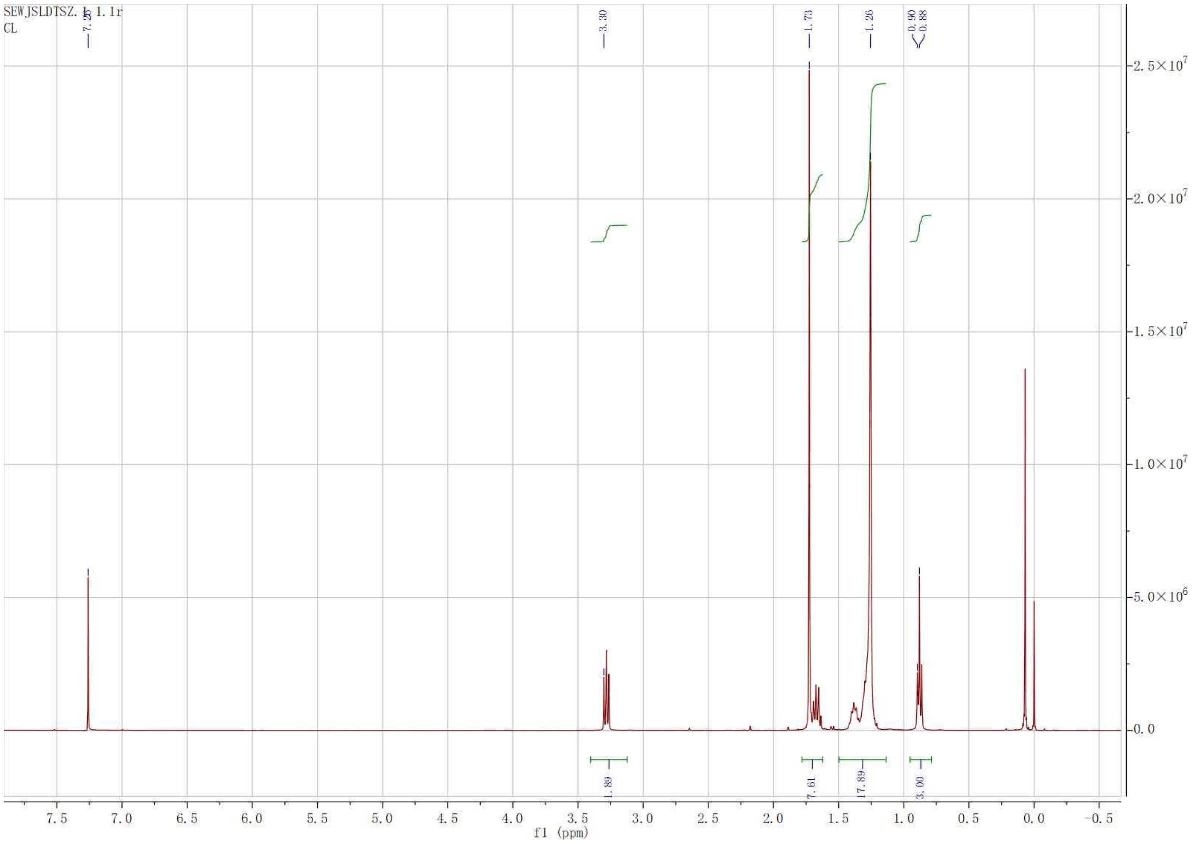

Figure S10. ^{1}H NMR spectrum of CTA.

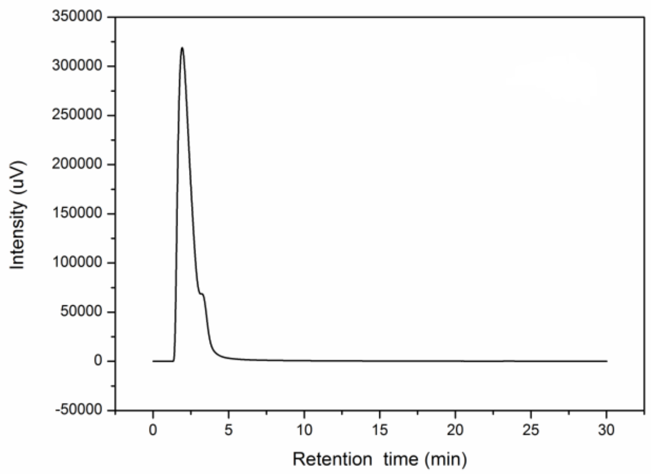

Figure S11. Chromatogram of mouse serum treated with poly(NIPAAm-*co*-DEAEMA-*co*-*t*BAAm)-grafted silica at 50 °C.

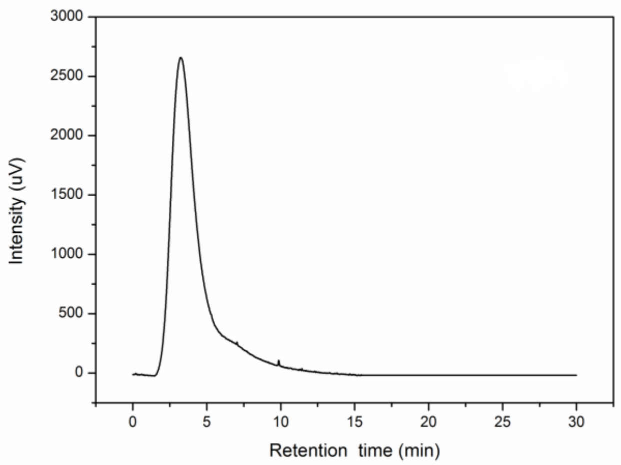

Figure S12. Chromatogram of group 2 treated with poly(NIPAAm-*co*-AAc-*co*-*t*BAAm)-grafted silica at 10 °C.

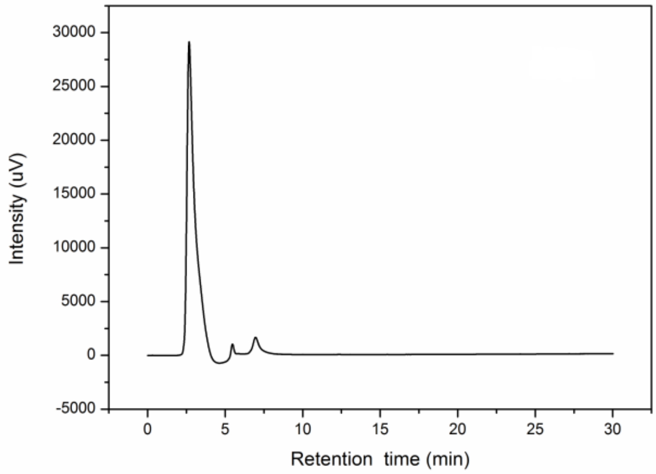

Figure S13. Chromatogram of group 4 treated with poly(NIPAAm-*co*-DMAEMA-*co*-VPBA) -grafted silica at 10 °C.

Table S1. The XPS of NH_2-grafted silica, CTA-grafted silica, poly(NIPAAm-*co*-DEAEMA-*co*-*t*BAAm)-grafted silica, poly(NIPAAm-*co*-AAc-*co*-*t*BAAm) -grafted silica and poly(NIPAAm-*co*-DMAEMA-*co*-VPBA)-grafted silica.

Element	C1s(%)	O1s(%)	N1s(%)	S2p(%)	B1s(%)
NH_2-grafted silica	39.61	58.94	1.45	0	0
CTA-grafted silica 30%	49.47	47.16	1.67	1.71	0
poly(NIPAAm-*co*-AAc-*co*-*t*BAAm)-grafted silica 30%	58.42	37.43	3.33	0.83	0
CTA-grafted silica 50%	46.50	49.68	2.37	1.44	0
poly(NIPAAm-*co*-AAc-*co*-*t*BAAm)-grafted silica 50%	74.40	22.78	2.18	0.64	0
poly(NIPAAm-*co*-DMAEMA-*co*-VPBA)-grafted silica 50%	68.80	26.51	2.54	0.82	1.33

Table S2. Type of proteins in crude mouse serum.

Number	Entry	Entry name	Number	Entry	Entry name
1	Q921I1	TRFE_MOUSE	109	Q8BPB5	FBLN3_MOUSE
2	P07724	ALBU_MOUSE	110	Q8BXA1	GOLI4_MOUSE
3	P20918	PLMN_MOUSE	111	P06336	IGHE_MOUSE
4	Q61838	PZP_MOUSE	112	P70665_3	SIAE_MOUSE
5	Q91X72	HEMO_MOUSE	113	Q6PGA0	RCOR3_MOUSE
6	P04186	CFAB_MOUSE	114	P42703	LIFR_MOUSE
7	Q00623	APOA1_MOUSE	115	P01898	HA10_MOUSE
8	Q61147	CERU_MOUSE	116	Q9JJZ2	TBA8_MOUSE
9	P08226	APOE_MOUSE	117	P34928	APOC1_MOUSE
10	O08677	KNG1_MOUSE	118	A1Z198	NL1B2_MOUSE
11	P01027	CO3_MOUSE	119	A2CFB8	A2CFB8_MOUSE
12	E9PV24	FIBA_MOUSE	120	Q8CI80	Q8CI80_MOUSE
13	P06909	CFAH_MOUSE	121	Q4FJS7	Q4FJS7_MOUSE
14	P26262	KLKB1_MOUSE	122	A0A075B5N9	A0A075B5N9_MOUSE
15	P28665	MUG1_MOUSE	123	P01845	LAC3_MOUSE
16	Q06890	CLUS_MOUSE	124	A0A075B5P4	A0A075B5P4_MOUSE
17	P21614	VTDB_MOUSE	125	Q3U679	Q3U679_MOUSE
18	Q61129	CFAI_MOUSE	126	Q8HWL1	Q8HWL1_MOUSE
19	Q91WP6	SPA3N_MOUSE	127	P02815	MUCL2_MOUSE
20	P29699	FETUA_MOUSE	128	B7ZNS9	B7ZNS9_MOUSE
21	Q9ESB3	HRG_MOUSE	129	A0A075B5P5	A0A075B5P5_MOUSE
22	Q9DBD0	ICA_MOUSE	130	Q99KA2	Q99KA2_MOUSE
23	Q8K182	CO8A_MOUSE	131	Q3UWC2	Q3UWC2_MOUSE
24	P11276	FINC_MOUSE	132	A0A1B0GQV5	A0A1B0GQV5_MOUSE
25	O88947	FA10_MOUSE	133	Q5M9K1	Q5M9K1_MOUSE
26	P07759	SPA3K_MOUSE	134	Q546G4	Q546G4_MOUSE
27	P13020	GELS_MOUSE	135	E0CXS6	E0CXS6_MOUSE
28	P01592	IGJ_MOUSE	136	Q3TCF3	Q3TCF3_MOUSE
29	P19221	THRB_MOUSE	137	Q544Y7	Q544Y7_MOUSE

30	Q8VCM7	FIBG_MOUSE	138	Q3TJ94	Q3TJ94_MOUSE
31	Q8K0E8	FIBB_MOUSE	139	Q3V1T9	Q3V1T9_MOUSE
32	O70362	PHLD_MOUSE	140	Q3UHS6	Q3UHS6_MOUSE
33	Q61703	ITIH2_MOUSE	141	H9H9R5	H9H9R5_MOUSE
34	Q07456	AMBP_MOUSE	142	E0CXN0	E0CXN0_MOUSE
35	P01029	CO4B_MOUSE	143	A0A1L1SRU8	A0A1L1SRU8_MOUSE
36	Q61730	IL1AP_MOUSE	144	H3BLC9	H3BLC9_MOUSE
37	Q8VCG4	CO8G_MOUSE	145	Q91X98	Q91X98_MOUSE
38	P01872_2	IGHM_MOUSE	146	Q3UEK9	Q3UEK9_MOUSE
39	Q61646	HPT_MOUSE	147	Q543J5	Q543J5_MOUSE
40	Q9QWK4	CD5L_MOUSE	148	Q3UUQ5	Q3UUQ5_MOUSE
41	P26039	TLN1_MOUSE	149	Q3UEK1	Q3UEK1_MOUSE
42	Q01339	APOH_MOUSE	150	Q8CFZ6	Q8CFZ6_MOUSE
43	P33587	PROC_MOUSE	151	Q3UAF7	Q3UAF7_MOUSE
44	P06683	CO9_MOUSE	152	Q4KL81	Q4KL81_MOUSE
45	Q61247	A2AP_MOUSE	153	Q9CVR0	Q9CVR0_MOUSE
46	Q9CQW3	PROZ_MOUSE	154	E9Q748	E9Q748_MOUSE
47	P00687	AMY1_MOUSE	155	Q3UHA5	Q3UHA5_MOUSE
48	P21180_2	CO2_MOUSE	156	Q3V2G1	Q3V2G1_MOUSE
49	P05366	SAA1_MOUSE	157	Q549A5	Q549A5_MOUSE
50	O88783	FA5_MOUSE	158	Q3TWK8	Q3TWK8_MOUSE
51	P41317	MBL2_MOUSE	159	Q3UAS4	Q3UAS4_MOUSE
52	O35664	INAR2_MOUSE	160	Q5ND36	Q5ND36_MOUSE
53	P03987	IGHG3_MOUSE	161	Q3UBS3	Q3UBS3_MOUSE
54	Q8BH35_2	CO8B_MOUSE	162	Q8K016	Q8K016_MOUSE
55	P33622	APOC3_MOUSE	163	A0A2I3BRQ3	A0A2I3BRQ3_MOUSE
56	P09813	APOA2_MOUSE	164	Q3UEL3	Q3UEL3_MOUSE
57	Q9R098	HGFA_MOUSE	165	Q64726	ZA2G_MOUSE
58	P47878	IBP3_MOUSE	166	Q6IRU2	TPM4_MOUSE
59	Q61268	APOC4_MOUSE	167	Q6PAC4	PCARE_MOUSE
60	P01942	HBA_MOUSE	168	Q6PEE2	CTIF_MOUSE
61	Q9DBB9	CPN2_MOUSE	169	Q566I6	Q566I6_MOUSE
62	Q01279	EGFR_MOUSE	170	F7B276	F7B276_MOUSE
63	Q9JHH6	CBPB2_MOUSE	171	A2A997	A2A997_MOUSE
64	P01878	IGHA_MOUSE	172	Q8R179	KBTB4_MOUSE
65	P12246	SAMP_MOUSE	173	Q8R3Q0	SARAF_MOUSE
66	P01654	KV3A1_MOUSE	174	Q9DAC2	Q9DAC2_MOUSE
67	P11758	HBB_MYOVE	175	Q54A77	Q54A77_MOUSE
68	Q8CG16	C1RA_MOUSE	176	I3QI43	I3QI43_MOUSE
69	P16301	LCAT_MOUSE	177	Q91X17	UROM_MOUSE
70	P20029	BIP_MOUSE	178	F6RCX2	F6RCX2_MOUSE
71	P52430	PON1_MOUSE	179	A0A1B0GS70	A0A1B0GS70_MOUSE
72	P01867_2	IGG2B_MOUSE	180	A0A2I3BPG0	A0A2I3BPG0_MOUSE
73	Q8BND5	QSOX1_MOUSE	181	B2RTL6	B2RTL6_MOUSE

74	Q8BFZ3	ACTBL_MOUSE	182	A0A075B5K2	A0A075B5K2_MOUSE
75	Q00897	A1AT4_MOUSE	183	A0A075B5N7	A0A075B5N7_MOUSE
76	O09164	SODE_MOUSE	184	A0A075B5T5	A0A075B5T5_MOUSE
77	P16882	GHR_MOUSE	185	A0A0A0UCD5	A0A0A0UCD5_MOUSE
78	Q9Z1R3	APOM_MOUSE	186	A0A0R4J1N3	A0A0R4J1N3_MOUSE
79	Q05020	APOC2_MOUSE	187	A2AEN9	A2AEN9_MOUSE
80	Q8BH61	F13A_MOUSE	188	A2AGP8	A2AGP8_MOUSE
81	Q61508	ECM1_MOUSE	189	A8DUQ1	A8DUQ1_MOUSE
82	P98064	MASP1_MOUSE	190	Q61287	Q61287_MOUSE
83	P01868	IGHG1_MOUSE	191	B8JJN0	B8JJN0_MOUSE
84	P05017_2	IGF1_MOUSE	192	D3YXF5	D3YXF5_MOUSE
85	O35930	GP1BA_MOUSE	193	Q9QXT7	Q9QXT7_MOUSE
86	Q9WVJ3	CBPQ_MOUSE	194	G3X9T8	G3X9T8_MOUSE
87	Q00896	A1AT3_MOUSE	195	Q3TR40	Q3TR40_MOUSE
88	P31532	SAA4_MOUSE	196	Q4V9W0	Q4V9W0_MOUSE
89	P01863	GCAA_MOUSE	197	Q6PIP8	Q6PIP8_MOUSE
90	E9Q414	APOB_MOUSE	198	Q61406	Q61406_MOUSE
91	P03388	ENV_MCFF3	199	Q61408	Q61408_MOUSE
92	P07309	TTHY_MOUSE	200	Q61876	Q61876_MOUSE
93	Q80YC5	FA12_MOUSE	201	Q64454	Q64454_MOUSE
94	Q61207	SAP_MOUSE	202	Q6GTX3	Q6GTX3_MOUSE
95	Q08879_2	FBLN1_MOUSE	203	Q6LD55	Q6LD55_MOUSE
96	Q80VP2	SPAT7_MOUSE	204	Q6S9I0	Q6S9I0_MOUSE
97	P01844	LAC2_MOUSE	205	Q6ZWX2	Q6ZWX2_MOUSE
98	P28798	GRN_MOUSE	206	Q91XL1	Q91XL1_MOUSE
99	P03953	CFAD_MOUSE	207	Q99LV9	Q99LV9_MOUSE
100	P26928	HGFL_MOUSE	208	Q9D1R7	Q9D1R7_MOUSE
101	Q9Z218	DPP6_MOUSE	209	Q9EQI5	Q9EQI5_MOUSE
102	P23953	EST1C_MOUSE	210	E9Q8N1	E9Q8N1_MOUSE
103	P29533	VCAM1_MOUSE	211	P06728	APOA4_MOUSE
104	P70186	EPYC_MOUSE	212	P29788	VTNC_MOUSE
105	Q7TMM9	TBB2A_MOUSE	213	Q8CG14	CS1A_MOUSE
106	P43025	TETN_MOUSE	214	Q61704	ITIH3_MOUSE
107	P01756	HVM12_MOUSE	215	Q00724	RET4_MOUSE
108	A6X935_2	ITIH4_MOUSE			

Table S3. Type of proteins in group 1

Number	Entry	Entry name	Number	Entry	Entry name
1	P07724	ALBU_MOUSE	1	P02535_2	K1C10_MOUSE
2	Q61838	PZP_MOUSE	2	Q91VD9	NDUS1_MOUSE
3	Q61147	CERU_MOUSE	3	P07361	A1AG2_MOUSE
4	Q61646	HPT_MOUSE	4	Q04447	KCRB_MOUSE
5	P01592	IGJ_MOUSE	5	O89020_3	AFAM_MOUSE

6	P23953	EST1C_MOUSE	6	B2RSH2	GNAI1_MOUSE
7	P01027	CO3_MOUSE	7	A2BIM8	MUP18_MOUSE
8	O08677	KNG1_MOUSE	8	B8JI96	B8JI96_MOUSE
9	Q00896	A1AT3_MOUSE	9	A0A075B5N2	A0A075B5N2_MOUSE
10	Q00623	APOA1_MOUSE	10	Q3TB63	Q3TB63_MOUSE
11	P21614	VTDB_MOUSE	11	Q3TP46	Q3TP46_MOUSE
12	Q9JJZ2	TBA8_MOUSE	12	A0A286YDH6	A0A286YDH6_MOUSE
13	Q8BFZ3	ACTBL_MOUSE	13	D3YTY9	D3YTY9_MOUSE
14	P07309	TTHY_MOUSE	14	Q542X9	Q542X9_MOUSE
15	P28665	MUG1_MOUSE	15	Q9DBN0	Q9DBN0_MOUSE
16	P29699	FETUA_MOUSE	16	Q80SX2	Q80SX2_MOUSE
17	E9PV24_2	FIBA_MOUSE	17	Q3TVS6	Q3TVS6_MOUSE
18	Q8VCM7	FIBG_MOUSE	18	B9EHT6	B9EHT6_MOUSE
19	A0A075B5N9	A0A075B5N9_MOUSE	19	Q4JFI8	Q4JFI8_MOUSE
20	Q99KA2	Q99KA2_MOUSE	20	Q791M3	Q791M3_MOUSE
21	Q3UWC2	Q3UWC2_MOUSE	21	Q3TUS2	Q3TUS2_MOUSE
22	Q5M9K1	Q5M9K1_MOUSE	22	Q91X22	Q91X22_MOUSE
23	Q546G4	Q546G4_MOUSE	23	Q3UA58	Q3UA58_MOUSE
24	Q3UEK9	Q3UEK9_MOUSE	24	Q791Q5	Q791Q5_MOUSE
25	Q543J5	Q543J5_MOUSE	25	Q3TF08	Q3TF08_MOUSE
26	Q3V2G1	Q3V2G1_MOUSE	26	Q3UJG0	Q3UJG0_MOUSE
27	Q549A5	Q549A5_MOUSE	27	Q3TXB7	Q3TXB7_MOUSE
28	Q5ND36	Q5ND36_MOUSE	28	Q3TDG1	Q3TDG1_MOUSE
29	Q3UBS3	Q3UBS3_MOUSE	29	Q8CI70	LRC20_MOUSE
30	Q3UEL3	Q3UEL3_MOUSE	30	Q3UEM7	Q3UEM7_MOUSE
31	A2A997	A2A997_MOUSE	31	Q05CL2	Q05CL2_MOUSE
32	A0A075B5N7	A0A075B5N7_MOUSE	32	Q6YJU1	Q6YJU1_MOUSE
33	G3X9T8	G3X9T8_MOUSE	33	Q3UGY5	Q3UGY5_MOUSE
34	Q3UAF7	Q3UAF7_MOUSE	34	A1L317	K1C24_MOUSE
35	Q9QWK4	CD5L_MOUSE	35	J3QPZ9	J3QPZ9_MOUSE
36	P06683	CO9_MOUSE	36	P56480	ATPB_MOUSE
37	Q9CQW3	PROZ_MOUSE	37	Q3V453	Q3V453_MOUSE
38	P33622	APOC3_MOUSE	38	Q8CGD9	Q8CGD9_MOUSE
39	P12246	SAMP_MOUSE	39	A0A1Y7VL93	A0A1Y7VL93_MOUSE
40	Q00897	A1AT4_MOUSE	40	D0ESZ4	D0ESZ4_MOUSE
41	P98064	MASP1_MOUSE	41	P0C6F1	DYH2_MOUSE
42	O35930	GP1BA_MOUSE	42	Q8R480	NUP85_MOUSE
43	Q9WVJ3	CBPQ_MOUSE	43	Q9EPL4	METL9_MOUSE
44	Q08879_2	FBLN1_MOUSE	44	P32261	ANT3_MOUSE
45	P29533	VCAM1_MOUSE	45	Q91WP0	MASP2_MOUSE
46	Q7TMM9	TBB2A_MOUSE	46	P35441	TSP1_MOUSE
47	P43025	TETN_MOUSE	47	Q8VCS0	PGRP2_MOUSE
48	P42703	LIFR_MOUSE	48	Q9D3H2	OBP1A_MOUSE
49	P11276	FINC_MOUSE	49	Q61897	KT33B_MOUSE

50	P07759	SPA3K_MOUSE	50	E9Q1Y9	E9Q1Y9_MOUSE
51	P13020	GELS_MOUSE	51	Q6IMF0	KRT87_MOUSE
52	Q4FJS7	Q4FJS7_MOUSE	52	Q8K0Y2	KT33A_MOUSE
53	Q8CI80	Q8CI80_MOUSE	53	Q8VCW2	K1C25_MOUSE
54	P01845	LAC3_MOUSE	54	Q9D312	K1C20_MOUSE
55	E0CXS6	E0CXS6_MOUSE			
56	Q3UEK1	Q3UEK1_MOUSE			
57	Q4KL81	Q4KL81_MOUSE			
58	Q6IRU2	TPM4_MOUSE			
59	Q566I6	Q566I6_MOUSE			
60	Q8R179	KBTB4_MOUSE			
61	A0A0R4J1N3	A0A0R4J1N3_MOUSE			
62	Q9QXT7	Q9QXT7_MOUSE			
63	Q3TR40	Q3TR40_MOUSE			
64	Q6GTX3	Q6GTX3_MOUSE			
65	Q6S9I0	Q6S9I0_MOUSE			
66	Q99LV9	Q99LV9_MOUSE			
67	Q9D1R7	Q9D1R7_MOUSE			
68	E9Q8N1	E9Q8N1_MOUSE			
69	Q3TWK8	Q3TWK8_MOUSE			
70	A0A075B5K2	A0A075B5K2_MOUSE			
71	Q6ZWX2	Q6ZWX2_MOUSE			
72	O35664	INAR2_MOUSE			

The red part represents the proteins of new identification compared with those of crude mouse serum.

Table S4. Type of proteins in group 3

Number	Entry	Entry name	Number	Entry	Entry name
1	P26262	KLKB1_MOUSE	1	Q9JJN5	CBPN_MOUSE
2	Q61129	CFAI_MOUSE	2	P46412	GPX3_MOUSE
3	Q61730	IL1AP_MOUSE	3	P39039_2	MBL1_MOUSE
4	Q8VCG4	CO8G_MOUSE	4	P01819	HVM43_MOUSE
5	Q01339	APOH_MOUSE	5	O09159	MA2B1_MOUSE
6	P21180_2	CO2_MOUSE	6	P18528	HVM57_MOUSE
7	Q8BH35_2	CO8B_MOUSE	7	Q3UR87	Q3UR87_MOUSE
8	Q61268	APOC4_MOUSE	8	Q6LAL7	Q6LAL7_MOUSE
9	P01942	HBA_MOUSE	9	Q8C8L1	Q8C8L1_MOUSE
10	P11758	HBB_MYOVE	10	Q711L0	Q711L0_MOUSE
11	P05017_2	IGF1_MOUSE	11	D3YY36	D3YY36_MOUSE
12	P31532	SAA4_MOUSE	12	Q3TBM1	Q3TBM1_MOUSE
13	P03388	ENV_MCFF3	13	Q3TMY3	Q3TMY3_MOUSE
14	P26928	HGFL_MOUSE	14	D3Z3I8	D3Z3I8_MOUSE
15	Q6PGA0	RCOR3_MOUSE	15	X5J5P3	X5J5P3_MOUSE
16	P34928	APOC1_MOUSE	16	Q922U2	K2C5_MOUSE

17	Q8HWL1	Q8HWL1_MOUSE	17	Q3TTY5	K22E_MOUSE
18	Q544Y7	Q544Y7_MOUSE	18	P48962	ADT1_MOUSE
19	H9H9R5	H9H9R5_MOUSE			
20	E0CXN0	E0CXN0_MOUSE			
21	E9Q748	E9Q748_MOUSE			
22	Q3UHA5	Q3UHA5_MOUSE			
23	Q6PAC4	PCARE_MOUSE			
24	Q8R3Q0	SARAF_MOUSE			
25	Q9DAC2	Q9DAC2_MOUSE			
26	I3QI43	I3QI43_MOUSE			
27	F6RCX2	F6RCX2_MOUSE			
28	Q6PIP8	Q6PIP8_MOUSE			
29	Q61406	Q61406_MOUSE			
30	Q61408	Q61408_MOUSE			
31	Q61876	Q61876_MOUSE			
32	Q9EQI5	Q9EQI5_MOUSE			
33	Q91X72	HEMO_MOUSE			
34	A0A075B5P5	A0A075B5P5_MOUSE			

The red part represents the proteins of new identification compared with those of crude mouse serum.

Table S5. Type of proteins in group 5

Number	Entry	Entry name	Number	Entry	Entry name
1	Q921I1	TRFE_MOUSE	1	P08607	C4BPA_MOUSE
2	P04186	CFAB_MOUSE	2	P34968	5HT2C_MOUSE
3	P06909	CFAH_MOUSE	3	P41233	ABCA1_MOUSE
4	Q9ESB3	HRG_MOUSE	4	P51885	LUM_MOUSE
5	O70362	PHLD_MOUSE			
6	Q61703	ITIH2_MOUSE			
7	P01029	CO4B_MOUSE			
8	P01872	IGHM_MOUSE			
9	P03987	IGHG3_MOUSE			
10	Q9R098	HGFA_MOUSE			
11	P06336	IGHE_MOUSE			
12	Q64726	ZA2G_MOUSE			
13	P70665	SIAE_MOUSE			
14	Q9Z218	DPP6_MOUSE			
15	P03953	CFAD_MOUSE			
16	O88783	FA5_MOUSE			
17	Q9DBB9	CPN2_MOUSE			
18	Q06890	CLUS_MOUSE			
19	P01878	IGHA_MOUSE			
20	Q9JHH6	CBPB2_MOUSE			
21	Q91X72	HEMO_MOUSE			

22	Q91X17	UROM_MOUSE

The red part represents the proteins of new identification compared with those of crude mouse serum.

Table S6. Type of proteins in group 6

Number	Entry	Entry name	Number	Entry	Entry name
1	P08226	APOE_MOUSE	1	E9Q1Y3	E9Q1Y3_MOUSE
2	Q91WP6	SPA3N_MOUSE	2	Q6DFV1	CNDG2_MOUSE
3	Q8K182	CO8A_MOUSE	3	A0A2K6EDJ7	A0A2K6EDJ7_MOUSE
4	O88947	FA10_MOUSE	4	P46460	NSF_MOUSE
5	P19221	THRB_MOUSE	5	P63038	CH60_MOUSE
6	Q07456	AMBP_MOUSE	6	A0A075B5V6	A0A075B5V6_MOUSE
7	P26039	TLN1_MOUSE	7	Q3UV17	K22O_MOUSE
8	P33587	PROC_MOUSE	8	D3Z7J9	D3Z7J9_MOUSE
9	Q61247	A2AP_MOUSE	9	D3Z6F5	D3Z6F5_MOUSE
10	P00687	AMY1_MOUSE	10	A0A140T8P3	A0A140T8P3_MOUSE
11	P41317	MBL2_MOUSE	11	P68040	RACK1_MOUSE
12	Q01279	EGFR_MOUSE	12	Q922J9	FACR1_MOUSE
13	P01654	KV3A1_MOUSE	13	Q9Z2H2	RGS6_MOUSE
14	Q8CG16	C1RA_MOUSE	14	Q9D0L7_2	ARM10_MOUSE
15	P16301	LCAT_MOUSE	15	Q3TAA6	Q3TAA6_MOUSE
16	P20029	BIP_MOUSE	16	H3BJV3	H3BJV3_MOUSE
17	P52430	PON1_MOUSE	17	Q547V2	Q547V2_MOUSE
18	P01867_2	IGG2B_MOUSE	18	H3BJV3	H3BJV3_MOUSE
19	O09164	SODE_MOUSE	19	A0A075B5P3	A0A075B5P3_MOUSE
20	P16882	GHR_MOUSE	20	A0A075B5K0	A0A075B5K0_MOUSE
21	Q9Z1R3	APOM_MOUSE	21	P02088	HBB1_MOUSE
22	Q05020	APOC2_MOUSE	22	Q9JJ59	ABCB9_MOUSE
23	Q8BH61	F13A_MOUSE	23	Q3UKU5	Q3UKU5_MOUSE
24	Q61508	ECM1_MOUSE	24	P52480	KPYM_MOUSE
25	E9Q414	APOB_MOUSE	25	P57774	NPY_MOUSE
26	Q80YC5	FA12_MOUSE	26	Q545J3	Q545J3_MOUSE
27	Q61207	SAP_MOUSE	27	E9PUE7	E9PUE7_MOUSE
28	Q80VP2	SPAT7_MOUSE	28	Q6PD31	TRAK1_MOUSE
29	P01844	LAC2_MOUSE	29	A2A6A1	GPTC8_MOUSE
30	P28798	GRN_MOUSE	30	Q3TGR2	Q3TGR2_MOUSE
31	P70186	EPYC_MOUSE	31	Q9ERV6	Q9ERV6_MOUSE
32	A6X935_2	ITIH4_MOUSE	32	B2RXW7	B2RXW7_MOUSE
33	Q8BPB5	FBLN3_MOUSE	33	A2NVX3	A2NVX3_MOUSE
34	Q8BXA1	GOLI4_MOUSE	34	Q9QWJ3	Q9QWJ3_MOUSE
35	P01898	HA10_MOUSE			
36	A1Z198	NL1B2_MOUSE			
37	A2CFB8	A2CFB8_MOUSE			
38	P02815	MUCL2_MOUSE			

39	B7ZNS9	B7ZNS9_MOUSE
40	Q3TJ94	Q3TJ94_MOUSE
41	Q3UHS6	Q3UHS6_MOUSE
42	A0A1L1SRU8	A0A1L1SRU8_MOUSE
43	H3BLC9	H3BLC9_MOUSE
44	Q91X98	Q91X98_MOUSE
45	Q9CVR0	Q9CVR0_MOUSE
46	Q3UAS4	Q3UAS4_MOUSE
47	A0A2I3BRQ3	A0A2I3BRQ3_MOUSE
48	Q6PEE2	CTIF_MOUSE
49	F7B276	F7B276_MOUSE
50	Q54A77	Q54A77_MOUSE
51	A0A1B0GS70	A0A1B0GS70_MOUSE
52	B2RTL6	B2RTL6_MOUSE
53	A2AEN9	A2AEN9_MOUSE
54	A2AGP8	A2AGP8_MOUSE
55	D3YXF5	D3YXF5_MOUSE
56	Q4V9W0	Q4V9W0_MOUSE
57	Q64454	Q64454_MOUSE
58	Q6LD55	Q6LD55_MOUSE
59	Q91XL1	Q91XL1_MOUSE
60	Q8CFZ6	Q8CFZ6_MOUSE
61	P20918	PLMN_MOUSE
62	Q3V1T9	Q3V1T9_MOUSE
63	P26262	KLKB1_MOUSE
64	Q01339	APOH_MOUSE
65	P47878	IBP3_MOUSE
66	Q3UUQ5	Q3UUQ5_MOUSE
67	A0A2I3BPG0	A0A2I3BPG0_MOUSE
68	A0A075B5T5	A0A075B5T5_MOUSE
69	A0A0A0UCD5	A0A0A0UCD5_MOUSE
70	Q61287	Q61287_MOUSE
71	Q921I1	TRFE_MOUSE
72	Q9DBD0	ICA_MOUSE
73	Q8K0E8	FIBB_MOUSE
74	Q8BND5	QSOX1_MOUSE
75	P01868	IGHG1_MOUSE
76	P01863	GCAA_MOUSE
77	P01756	HVM12_MOUSE
78	Q00897	A1AT4_MOUSE
79	Q9WVJ3	CBPQ_MOUSE
80	E9Q8N1	E9Q8N1_MOUSE
81	A0A075B5N7	A0A075B5N7_MOUSE
82	A0A075B5N9	A0A075B5N9_MOUSE

83	P06728	APOA4_MOUSE
84	P29788	VTNC_MOUSE

The red part represents the proteins of new identification compared with those of crude mouse serum.
